# Gene Network in Fruit Flies Guides Nervous System Repair

**DOI:** 10.1371/journal.pbio.1001136

**Published:** 2011-08-30

**Authors:** Janelle Weaver

**Affiliations:** Freelance Science Writer, Glenwood Springs, Colorado, United States of America

**Figure:**
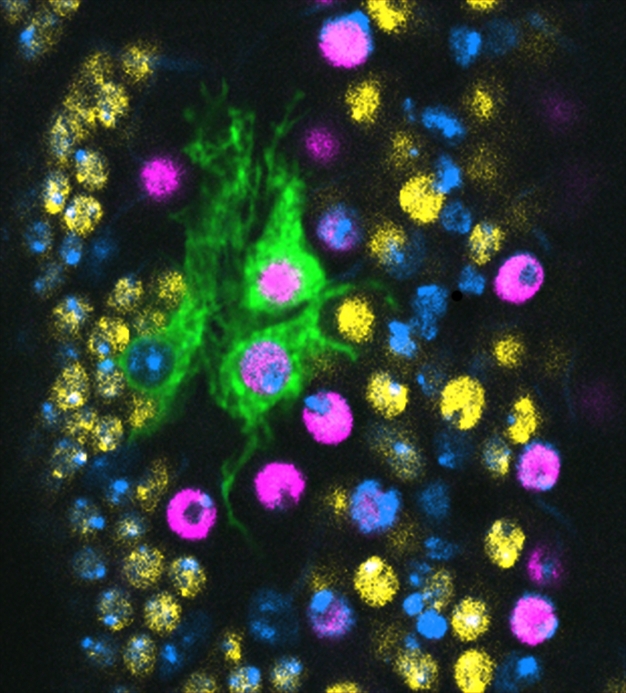
Interface Glia of the *Drosophila* larval ventral nerve cord normally enwrap axons and can divide after injury to promote repair, enabled by the genes *prospero* and *Notch*. Mitotic recombination clone of Interface Glia (green) and Prospero (magenta). All nuclei are labeled with DAPI (blue), neuronal nuclei with anti-Elav (yellow). Image credit: Kentaro Kato.

Animals as distinct as fruit flies and humans can repair and replace cells in their nervous system after damage caused by injuries or diseases. To support healing, cells called glia increase in number near damaged areas, engulf cellular debris, and restore neuronal function by wrapping around axons, long projections that conduct electrical impulses. Cell growth must be tightly regulated during regeneration so that tumors don't develop, but how genes orchestrate this process has not been explained by past studies.

In the current issue of *PLoS Biology,* a research team led by Alicia Hidalgo, a neuroscientist at the University of Birmingham in the United Kingdom, reveals a gene network that underlies glial responses to injury in the central nervous system of the fruit fly (*Drosophila melanogaster*). This network consists of two feedback loops that promote regeneration and repair, but then put the brakes on cell division to prevent uncontrolled growth.

Through a series of genetic experiments, the team found that a protein called Prospero engages in a tug-of-war with two cell signaling pathways, Notch and Dorsal/NF-kappaB. Prospero inhibits cell proliferation, while Notch and Dorsal/NF-kappaB activate it. Because these opposing signals boost each other's activity, they keep glia on the brink of dividing under normal conditions.

But when the researchers stabbed the ventral nerve cord of flies with a needle, glia became more abundant close to the wounds. Raising levels of Prospero through genetic modifications resulted in fewer glia after injury, but boosting either Notch or Dorsal/NF-kappaB signaling intensified glial proliferation.

Next, the researchers tested what happens when the negative feedback breaks down. Disrupting the balance between the opposing signals by enhancing either Notch or Dorsal/NF-kappaB signaling in mutant flies lacking Prospero resulted in tumor-like overgrowths.

The interplay between Notch and Prospero also regulates regeneration and repair. A surge in Notch signaling caused wounds to shrink and glia to encircle damaged axons, and Prospero enabled the clearance of cellular debris. But lesions were larger in flies with no Prospero, too much Prospero, decreased Notch activity, or a deficiency in Prospero combined with overstimulation of the Notch pathway.

This gene network may allow cells to adapt to changes in their environment during the normal course of development and throughout life, thus preserving the stability of the nervous system. Through these pathways, cells could multiply or form connections during learning without disturbing the brain's structure and function.

In addition to potentially explaining how the nervous system maintains equilibrium, this research could lead to new therapeutic strategies for regeneration in the adult mammalian central nervous system, which does not undergo extensive repair. If this gene network exists in humans, it might be possible to manipulate the genes and transplant cells into patients with spinal cord injuries or certain neurological disorders.

**Kato K, Forero MG, Fenton JC, Hidalgo A (2011) The Glial Regenerative Response to Central Nervous System Injury Is Enabled by Pros-Notch and Pros-NFκB Feedback. doi:10.1371/journal.pbio.1001133**

